# Quercetin positively affects gene expression profiles and metabolic pathway of antibiotic-treated mouse gut microbiota

**DOI:** 10.3389/fmicb.2022.983358

**Published:** 2022-08-25

**Authors:** Wei Mi, Zhiyong Hu, Lanlan Xu, Xiangyu Bian, Wu Lian, Shuying Yin, Shuying Zhao, Weina Gao, Changjiang Guo, Tala Shi

**Affiliations:** ^1^Department of Nutrition and Food Hygiene, School of Public Health and Management, Binzhou Medical University, Yantai, China; ^2^Tianjin Institute of Environmental and Operational Medicine, Tianjin, China

**Keywords:** quercetin, gut microbiota, gene expression, metagenomics, bacterial species

## Abstract

Quercetin has a wide range of biological properties that can be used to prevent or decrease particular inflammatory diseases. In this study, we aimed to investigate the gene expression profile and metabolic pathway of the gut microbiota of an antibiotic-treated mouse model administered quercetin. Blood, feces, and intestinal tissue samples were collected and metagenomic sequencing, enzyme-linked immunosorbent assay, and western blot analysis were used to detect variations. The results showed that the quercetin-treated group exhibited increased levels of health beneficial bacterial species, including *Faecalibaculum rodentium* (103.13%), *Enterorhabdus caecimuris* (4.13%), *Eggerthella lenta* (4%), *Roseburia hominis* (1.33%), and *Enterorhabdus mucosicola* (1.79%), compared with the model group. These bacterial species were positively related to butyrate, propionate, and intestinal tight junction proteins (zonula occludens-1 and occludin) expression, but negatively related to serum lipopolysaccharide and tumor necrosis factor-α level. In addition, the metabolic pathway analysis showed that dietary quercetin significantly enhanced spliceosomes (111.11%), tight junctions (62.96%), the citrate cycle (10.41%), pyruvate metabolism (6.95%), and lysine biosynthesis (5.06%), but decreasing fatty acid biosynthesis (23.91%) and *N*-glycan (7.37%) biosynthesis. Furthermore, these metabolic pathway changes were related to relative changes in the abundance of 10 Kyoto Encyclopedia of Genes and Genomes genes (K00244, K00341, K02946, K03737, K01885, k10352, k11717, k10532, K02078, K01191). In conclusion, dietary quercetin increased butyrate-producing bacterial species, and the acetyl-CoA-mediated increased butyrate accelerated carbohydrate, energy metabolism, reduced cell motility and endotoxemia, and increased the gut barrier function, thereby leading to healthy colonic conditions for the host.

## Introduction

Disturbances of the gut microbiota could damage barrier function and a loss of the intestinal barrier causes systemic immune activation, resulting in a wide range of extra intestinal autoimmune and inflammatory diseases (Takiishi et al., 2017; Wang et al., 2020). Gut microbial fermentation metabolites, communities, and their metabolic pathway or associated genes are believed to play a deterministic role in human health and disease. However, manipulation of the gut microbiota with diet, probiotics, or prebiotics have been shown to improve human health (Gill et al., 2001; Clements, 2018). Dietary polyphenols are important bioactive components found in fruits and vegetables that are known to be potent antioxidants, and can bind to transitional metal ions and can improve the mucus layer, villus morphology, and tight junction protein levels in mice (D’Andrea, 2015; Nabavi et al., 2015; Wang et al., 2020). Quercetin is a representative polyphenolic flavonoid that gained significant attention for its ability to inhibit diseases such as cardiovascular disease, inflammation, cancer, diabetes, and obesity (Etxeberria et al., 2015; Nie et al., 2019). Research has revealed that the beneficial bioactivity of quercetin is primarily due to its metabolization in the intestines and liver in various naturally occurring conjugated isoforms following its metabolism, whereby the products of quercetin are absorbed and extensively distributed in host tissues (Graefe et al., 2001; Chen et al., 2005; de Boer et al., 2005).

In addition, the functional properties and underlying mechanisms of action of quercetin have become the focus of increasing attention due to its interaction with the gut microbiota and their metabolites. Quercetin supplementation in high-fat diet-fed mice was shown to greatly affect different taxonomic groups in the gut microbiota composition, counteracting its dysbiosis through modulation of lipid metabolism-related gene expression (Porras et al., 2017). Yang et al. reported that quercetin increased *microRNA-219* (*miR-219*), *miR-15a*, and *miR-132* expression to inhibit phosphorylated-extracellular signal-related kinase 1 and 2 (p-ERK1/2) and tau phosphorylation (Yang et al., 2020).

Furthermore, through this action, quercetin induces species of the *Tenericutes* and *Proteobacteria* phyla to elevate butyric acid production in the cecum, thereby reversing the cognitive dysfunction induced by dietary advanced glycation products (Yang et al., 2020). Ahn et al. reported that quercetin suppressed lipogenesis by inhibiting the gene expression levels of fatty acid synthase and key adipogenic factors (peroxisome proliferator-activated receptor [PPAR]-γ and CCAAT/enhancer-binding protein [C/EBP]-α and the activity of acetyl-CoA carboxylase (Ahn et al., 2008; Zhao et al., 2017). We previously found that quercetin effectively recovered the gut microbiota function in antibiotic-treated mice, increased the intestinal villi length and mucosal thickness, and acted as a prebiotic in combatting gut dysbiosis (Shi et al., 2020). However, the effect of quercetin on the phenotype and gene expression of the resident gut bacteria, and on microbial metabolic transformation to functional connections are not well understood.

The investigation of the biochemical functions of microbial species and their metabolites represents a promising opportunity to bridge the knowledge gap between gut bacteria and their effect on host metabolism. Metagenomic sequencing was used to overcome the defect of 16S rRNA, by increasing the predictive ability of genes, enhancing the detection of microbial species, and allowing the detailed descriptions of the functions of the identified genes. Furthermore, this technique could enable the acquisition of genetic information regarding the entire community of microorganisms (Zou et al., 2020). In this study, we used metagenomics to elucidate the effects of quercetin on the metabolic pathway, composition, function, and gene expression recovery of the gut microbiota. Further, our findings revealed the mechanism of action of quercetin on the gut microbiota and host health.

## Materials and methods

### Animals, diets, and experimental model

The 60 C57BL/6J mice obtained from the Experimental Animal Center of the Chinese Academy of Military Medical Sciences (Tianjin, China) were housed at 23°C ± 1°C and 40–60% relative humidity, under a 12-h light/dark cycle. Furthermore, the mice were allowed *ad libitum* access to water and were adaptively fed an AIN-93G diet for 5 days under these conditions. Then, the mice were randomly divided into two groups, control (AC, *n* = 20) and model (AA, *n* = 40) groups, according to body weight. The AC and AA groups were intragastrically administered 0.5 mL each of phosphate-buffered saline (PBS) and the antibiotic cocktail (0.5 g/L vancomycin, 1 g/L neomycin sulfate, 1 g/L metronidazole, 1 g/L ampicillin), respectively, once daily for 7 days to disrupt the gut microbial balance.

Then, samples of blood, feces, and transverse colon tissue were collected from four mice from each group. After successfully establishing the disrupted gut microbiota model, the AC group was designated as TC and the AIN-93G diet was continued, whereas the AA group was further divided into two subgroups, normal AIN-93G (TA, *n* = 18) and 0.2% quercetin-containing AIN-93G (TQ, *n* = 18) fed groups, which were fed the diets for another 14 days. At the end of the experiment, blood samples were collected from the orbital vein of each mouse, centrifuged (15,000 rpm, 10 min), and the serum was then stored at −20°C. Feces sample were collected from the rectum under clean bench conditions, snap-frozen in liquid nitrogen, and then stored at −80°C for further study. The chart of experimental protocol can be found in Supplementary Figure 1.

### Metagenomic analyses

The metagenomic sequencing method used to investigate functional changes was performed using an Illumina HiSeq 2500 sequencing system (Illumina, San Diego, CA, United States). DNA was extracted from feces samples from the TC, TA, and TQ groups and a > 2 μg sample was used to construct the library using the NEBNext Ultra DNA Library Prep kit for Illumina), following the manufacturer’s protocol. Size selection of adaptor-ligated DNA was performed using AxyPrep Mag polymerase chain reaction (PCR) clean-up (Axygen) and fragments of ∼410 bp were recovered. Each sample was then amplified using PCR run for eight cycles using P5 (AGATCGGAAGAGCGTCGTGTAGGGAAAGAGTGT) and P7 primers (AGATCGGAAGAGCACACGTCTGAACTCCAGT CAC).

The PCR products were cleaned-up using AxyPrep Mag PCR clean-up, validated using an Agilent 2100 bioanalyzer (Agilent Technologies, Palo Alto, CA, United States), and quantified using Qubit2.0 Fluorometer (Invitrogen, Carlsbad, CA, United States). Next, libraries with different indexes were multiplexed and loaded on an Illumina HiSeq instrument according to the manufacturer’s instructions. Sequencing was conducted using a 2 × 150 paired-end (PE) configuration, whereas the image analysis and base calling were conducted using the HiSeq control software (HCS) + OLB + GAPipeline-1.6 (Illumina) using the HiSeq sequencing system.

### Data analysis

Raw sequencing reads were trimmed using cutadapt (v1.9.1), low-quality, N-rich, adapter-polluted, and host-contaminated reads were removed. Samples were each assembled *de novo* separately and the best assembly result of the scaffold, which had the largest N50, was selected for the subsequent analysis. CD-HIT was used to cluster shafts derived from the assembly with a default identity of 0.95. To analyze the relative abundance of scaftigs in each sample, PE clean reads were mapped to assembled scaftigs using the Burrows–Wheeler Aligner (BWA, version 0.7.12) to generate read coverage information for assembled scaftigs. The corresponding shafts were mapped to the mass of bacteria extracted from the nucleotide (NT) database of the National Center for Biotechnology Information (NCBI).

The lowest common ancestor (LCA) algorithm (applied using the MEGAN software system) was used to ensure the annotation significance by picking out the classified LCA for final display. Genes were predicted using MetaGeneMark, whereas the protein Basic Local Alignment Search Tool (BLASTP) was used to search the protein sequences of the predicted genes in the non-redundant (NR), Carbohydrate-Active enZYmes (CAZy), evolutionary genealogy of genes: Non-supervised Orthologous Groups (eggNOG), and Kyoto Encyclopedia of Genes and Genomes (KEGG) databases with an E value < 1e-5. To determine the similarity or difference of taxonomic and functional components between different samples, relative clustering analysis and principal component analysis (PCA), LEfSe were performed.

### Intestinal permeability and inflammatory function

The gut mucosal permeability indexes, lipopolysaccharides (LPS), and intestinal inflammatory factors including interleukin (IL)-10, tumor necrosis factor (TNF)-α, and interferon (IFN)-γ were measured using an enzyme-linked immunosorbent assay (ELISA) kit (Wuhan ColorfulGene Biological Technology, Wuhan, China). The large intestinal samples (200 mg) collected from TC, TA, TQ, were homogenized, centrifuged (15,000 rpm, 10 min, 4°C), the collected supernatant and serum sample was diluted five times, and then analyzed using the ELISA kit, strictly following the manufacturer’s protocol.

### Intestinal tight junction protein expression

The intestinal tight junction-related proteins zonula occludens (ZO)-1 and occludin were analyzed. Intestinal tissue samples from the mice were homogenized in protein extraction solution and protein concentrations of the homogenates were determined using a bicinchoninic (BCA) protein assay kit (Thermo Scientific, United States). The protein was diluted with ultra-pure water to 2 μg/μL, mixed with loading buffer at a ratio of 4:1, boiled for 5 min, cooled to 20°C, and stored at −40°C. Then, the protein homogenate (10 μL) was separated using 10% sodium dodecyl sulfate-polyacrylamide gel electrophoresis (SDS-PAGE) at a constant voltage of 80 V. The separated proteins were transferred onto a polyvinylidene fluoride (PVDF) membrane (Bio-Red), blocked with 5% skim milk in tris-buffered saline (TBS), and incubated overnight with appropriate primary antibodies at 4°C.

The immunoblots were washed three times in TBS buffer and incubated with appropriate secondary antibodies for 1 h. After washing the membranes several times, the immunoblotted proteins were visualized using an enhanced chemiluminescence (ECL) western bolting luminal reagent, and the band intensities were quantified using the Gel-Pro 6.0 software analyzer. The results were expressed as the ratio of the optical density of the target protein to that of β-actin. The following antibodies used in this experiment: anti-β-actin (Cat#4970), anti-rabbit immunoglobulin G [IgG] horseradish peroxidase (HRP)-linked (Cat#7074; both from, Cell Signaling Technology), anti-occludin (Cat#ab216327), and anti-ZO-1 tight junction protein (Cat#ab216880, both from Abcam).

### Statistical analysis

The results of experiments conducted with two groups were analyzed using an unpaired Student’s *t*-test, and those with more than two groups were analyzed using a one-way analysis of variance (ANOVA) followed by Tukey’s *post hoc* test or the Dunn test to further compare the groups. Data are presented as the means ± standard error of the mean (SEM) and results with a *P*-value < 0.05 were considered statistically significant.

## Results

### Antibiotic cocktail disrupted gut microbial composition

**FIGURE 1:**
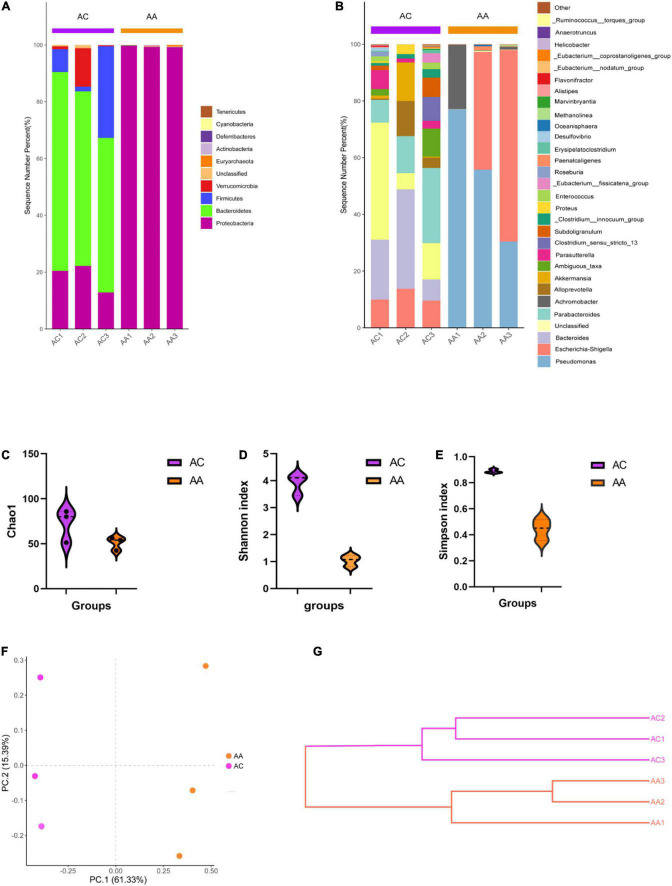
Antibiotic cocktail treatment effects on gut microbial diversity. **(A)** Phylum level gut microbial structure; **(B)** Genus level gut microbial composition; **(C–E)** Alpha diversity analysis; **(F,G)** beta diversity analysis.

The 16S rRNA sequencing results showed varying microbial profiles at different classification levels. Consist with our previous study (Bian et al., 2022), antibiotic cocktail treated group (AA) decreased all bacteria except Proteobacteria phylum, compared with control (AC) (Figure 1A). At the genus level, in the AA group, all bacteria genera decreased, except for *Pseudomonas*, *Escherichia-Shigella*, and *Achromobacter*, (Figure 1B). In addition, significantly decreased Shannon, Simpson and Chao1 indices were obtained in the AA group, indicating a reduction of microbial richness (Figures 1C–E). The PCoA and weighted UniFrac tree beta diversity analysis also clearly clustered samples (Figures 1F,G). These results indicate that the abundance and composition of intestinal microbial were significantly reduced by intragastric administration of antibiotic cocktails, inferring that under our experimental condition, we successfully established a gut microbial disrupted mouse model.

### Quercetin induced gut microbial changes

**FIGURE 2:**
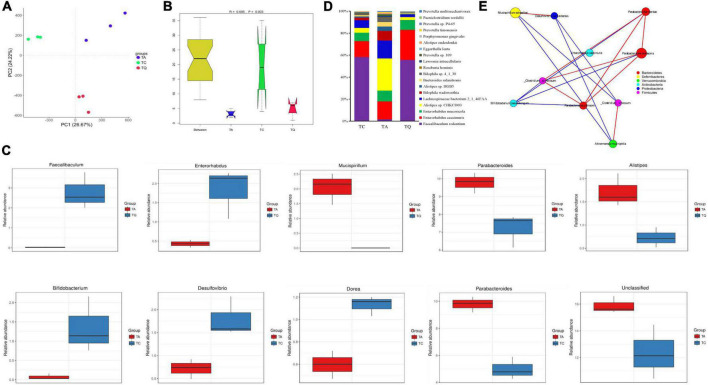
Gut microbial diversity after quercetin treatment. **(A)** Principle coordinate analysis of gut microbial structure; **(B)** Anosim analysis of gut microbial structure within groups; **(C)** Top 5 Significantly different bacteria genera between groups; **(D)** Significantly different bacteria species between TA and TQ; **(E)** Spearman correlation analysis of bacteria species, which LDA score higher than 4, red line indicate positive correlation, blue line indicate negative correlation.

Quercetin treatment altered the gut microbiota composition of antibiotic-treated mice, from phylum to species, the results was illustrated using the Krona flat pattern. The three groups were clearly separated in the PCA with significant [correlation coefficient (r) = 0.695, *P* = 0.003] differences within groups according to the analysis of similarities (Anosim, Figures 2A,B). The Metastats analysis showed the top five significantly different bacterial genera between groups (Figure 2C). Compared with TC, TA increased the relative abundance of *Parabacteroides*, decreased the *Dorea*, *Desulfovirio*, *Bifidobacterium*, and *unclassified* bacteria genera. Compared with TA, TQ increased the relative abundance of *Faecalibaculum*, *Enterohabdus*, decreased the *Parabacteroides* and *Alistipes* and *Musicspirillum* genera. The difference in the abundance of taxa within groups was also identified using the linear discriminant analysis (LDA) effect size (LEfSe) method using non-parametric factorial Kruskal–Wallis and Wilcoxon rank-sum tests. The histogram of the distribution of LDA values (> 2) shows the typical microbiota of the groups (Supplementary Figures 2A,B). The TQ group showed selective enrichment of *Verrucomicrobia*, *Akkermansia*, and *Enterohabdus* phyla, whereas the TA group revealed particular effects on the *Parabacteroides* and *Alistipes* genera and the *Rikenellanceae* family, and the TC group had a high abundance of the *Mucispirillum* genus and *Deferribacteres* family.

The results showed that 2,104 bacterial species were annotated, and the ANOVA determined that 139 species were significantly different within groups (*P* < 0.05). Compared with the TC group, the TA group showed a significant increase (*P* < 0.05) in the levels of 25 bacteria species including *Parabacteroides distasonis, Bacteroides sp. 2_1_33B*, and *Clostridiales bacterium VE202-1*6, whereas levels of 21 bacterial species were decreased including *Bifidobacterium pseudopodium*, *Eubacterium plexicaudatum*, and *Dorea sp. CAG_105* (Supplementary Table 1). However, compared with the TA group, the TQ group showed a significantly (*P* < 0.05) higher relative abundance of nine bacterial species (*P* < 0.05) including *Faecalibaculum rodentium* (103.13%), *Enterorhabdus caecimuris* (4.13%), *Eggerthella lenta* (4%), *Roseburia hominins* (1.33%), and *Enterorhabdus mucosicola* (1.79%).

Furthermore, there was a significant reduction in the abundance of 11 bacteria species (*P* < 0.05), such as *Alistipes sp. CHKCI003* (0.73%), *Alistipes onderdonkii* (0.8%), *Bacteroides salanitronis* (0.55%), *Lachnospiraceae bacterium 2_1_46FAA* (0.59%), and *Lawsonia intracellularis* (0.75%, Figure 2D and Supplementary Table 1). The LDA (> 4) showed that *Akkermansia muciniphila*, *Mucispirillum schaedleri*, and *P. distasonis* were the main biomarkers for TQ, TC, and TA, respectively (Supplementary Figure 2C). The results of the Spearman correlation network analysis of the LDA score > 4 of the bacterial species showed that *A. muciniphila* and *Bifidobacterium pseudolongum* were negatively related to *Parabacteroides johnsonii* (Figure 2E).

### Quercetin changed metabolic pathway of gut microbiota

**FIGURE 3:**
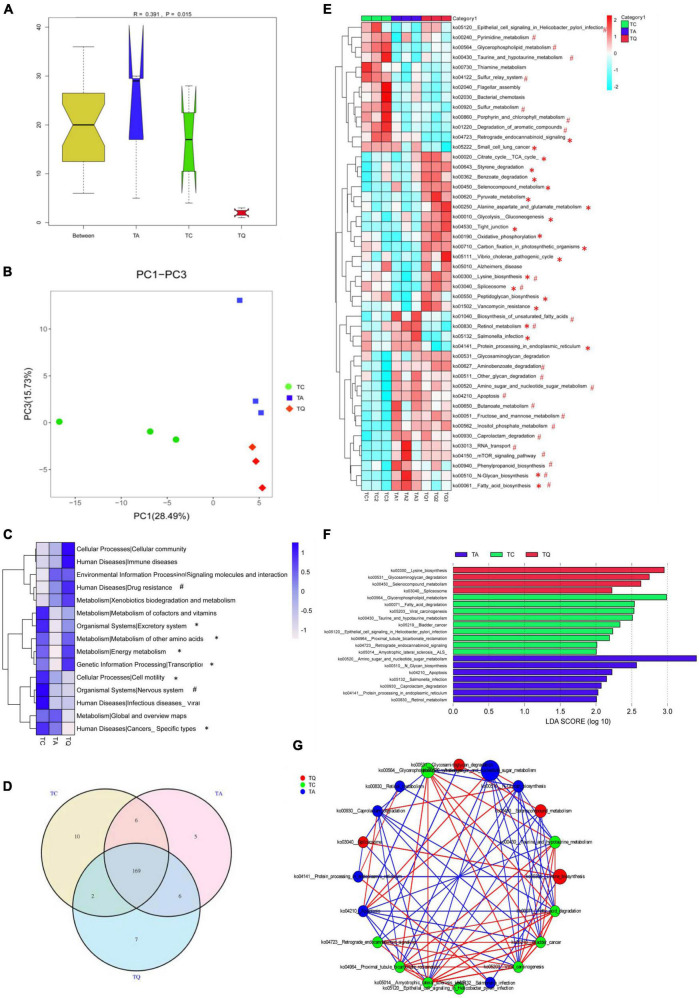
Metabolic pathway changes of gut microbiota after quercetin treatment. **(A)** Anosim analysis of KEGG metabolic pathway within groups; **(B)** Principle coordinate analysis of KEGG metabolic pathway; **(C)** KEGG level 2 differences within groups, *Significantly different between TA-TQ, # significantly different between TC-TA; **(D)** Venn diagram of KEGG metabolic pathway within three groups; **(E)** 47 significantly different pathways within group; *Significantly different between TA-TQ, # significantly different between TC-TA; **(F)** LDA analysis of 47 significantly different bacteria species; **(G)** Spearman correlation network analysis of LDA score higher than 4 pathways, different circle size indicate their relative abundance, different color line indicate different correlation, red line indicate positive correlation, blue line indicate negative correlation.

Metagenomic sequencing was additionally used for functional analysis and 611,264 Unigene sequences (Supplementary Table 2) were predicted in our study. The Anosim results showed that the KEGG orthologous (KO) genes were separated into groups (*P* = 0.015, Figure 3A). Principal coordinate analysis (PCoA) based on KEGG modules revealed differences in microbial functions within groups (Figure 3B). The number of carbohydrate metabolism-related genes was highest at KEGG level 1. Based on the KEGG level 2 analysis, the TA group demonstrated a higher level of drug resistance, but lower amino acid and energy metabolism, as well as nervous system effects than the TC group. However, the TQ group showed higher levels of amino acid and energy metabolism, transcription, and excretory system activity, with lower incidences of specific cancers and cell motility relative abundance than the TA group (Figure 3C).

At KEGG level 3, the Venn diagram illustrates that the TC, TA, and TQ groups had 10, 5, and 7 unique pathways, respectively, and 169 common metabolic pathways (Figure 3D). The one-way ANOVA showed that 47 pathways were significantly different within groups (Figure 3E). Compared with the TC group, the TA group showed significantly lower levels (*P* < 0.05) in the activity of 10 metabolic pathways, including spliceosome (ko03040, 43.75%), sulfur metabolism (ko00920, 23.20%), lysine biosynthesis (ko00300, 2.9%), and glycerophospholipid metabolism (ko00564, 10.02%). In contrast, 15 metabolic pathways showed higher activity in the TA group than in the TC group, including amino sugar and nucleotide sugar metabolism (ko00520, 6.73%), *N*-glycan biosynthesis (ko00510, 14.61%), fatty acid biosynthesis (ko0006132.56%), and retinol metabolism (ko00830, 40%). However, compared with the TA group, the TQ group showed a significantly lower level (*P* < 0.05) of activity in seven pathways, including fatty acid biosynthesis (ko00061, 23.91%), *N*-glycan biosynthesis (ko00510, 7.37%), retinol metabolism (ko00830, 75%), *Salmonella* infection (ko05132, 19.35%), increased 15 kinds pathways including Spliceosome (ko03040, 111.11%), tight junction (ko04530, 62.96%), citrate cycle (ko00020, 10.41%), and lysine biosynthesis (ko00300, 5.06%; Figure 3E). The LDA score analysis determined that the most effective pathways for TC, TA, and TQ were glycerophospholipid metabolism, amino sugar and nucleotide sugar metabolism, and lysine biosynthesis, respectively (Figure 3F). The LDA biomarker pathways correlation analysis showed that lysine biosynthesis was positively correlated to glycerophospholipid metabolism but negatively related to amino sugar and nucleotide sugar metabolism (Figure 3G).

### Quercetin changed gene expression profiles of gut microbiota

**FIGURE 4:**
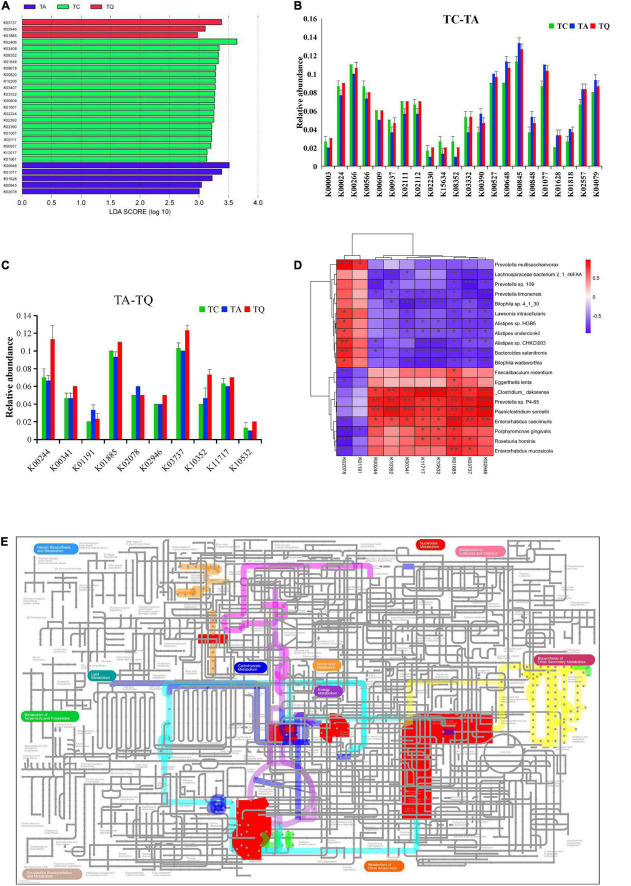
Quercetin effects on gene expression profile of gut microbial. **(A)** LDA analysis of 80 genes which annotated on significantly different metabolic pathways; **(B)** Significantly different genes between TC and TA; **(C)** Significantly different genes between TA and TQ; **(D)** Spearman correlation analysis of 10 genes and significantly different bacteria species between TA and TQ; **(E)** iPath diagram of 10 genes and their related metabolic pathway, different color line indicate different metabolic pathway.

Based on the KEGG annotation analysis, we identified 6,258 genes from nine samples, three groups shared 2,180 genes, and the one-way ANOVA showed that 272 genes were significantly different within groups, whereas 80 of 272 genes were expressed in 47 significantly different pathways. The LDA showed that k02406 (*fliC*, flagellin), K00648 (*fabH*, 3-oxoacyl-acyl-carrier-protein synthase III), k03737 (*por*, *nifJ*, flavodoxin oxidoreductase) were the predominant genes of the TC, TA, and TQ groups (Figure 4A). Furthermore, the Dunn test of 80 genes showed that compared with the TC group, in the TA group, the relative abundance of 10 genes was upregulated, whereas this was downregulated for 12 others (Figure 4B).

However, compared with the TA group, the relative abundance of the following eight genes in the TQ group k10532 (*HGSNAT*), K00244 (*frdA*), k10352 (*MYH*), k00341 (*null*), k02946 (*RP-S10*, *MRPS10*, reps), k03737 (*por*, *nifJ*), k01885 (*EARS*), and k11717 (*sufS*) was upregulated at 100%, 70%, 57.14%, 28.57%, 25%, 23.33%, 17.86%, and 16.67%, respectively. Furthermore, the abundance of two genes k01191 (E3.2.1.24) and k02078 (*acpP*), was downregulated at 30% and 16.67%, respectively (Figure 4C).

The 10 genes and implicated pathways are shown on the iPath diagram (Figure 4D), and are related to glycolysis/gluconeogenesis, citrate [tricarboxylic acid (TCA)] cycle, pyruvate metabolism, butanoate metabolism, oxidative phosphorylation, porphyrin and chlorophyll metabolism, RNA transport pathway, fatty acid biosynthesis, and other glycan degradation pathway. Moreover, the results of the Spearman correlation analysis of 10 genes and significantly different gut microbial species determined eight upregulated genes positively related to *E. caecimuris*, the bacteria identified as a biomarker species in the TQ group (Figure 4E).

**FIGURE 5:**
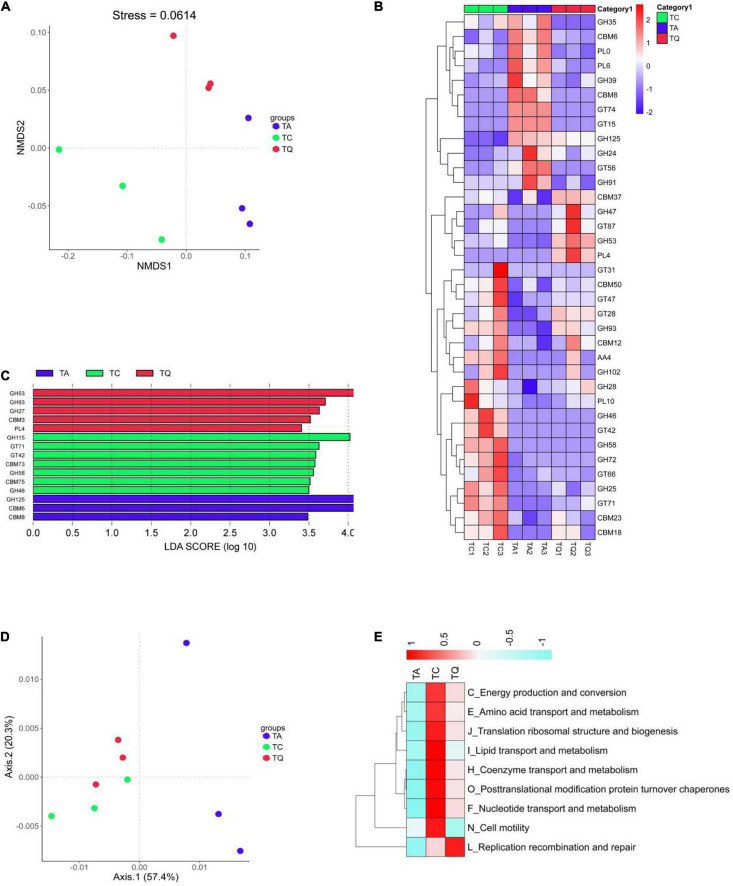
Functional analysis based on CAZy orthologs and egg NOG Orthologous. **(A)** NMDS analysis of CAZy function was clearly separated within groups; **(B)** Significantly different enzymes within groups; **(C)** LDA analysis of significantly different enzymes; **(D)** PCoA analysis of NOGs function was clearly separated within groups; **(E)** NOGs based significantly different metabolic pathway within groups.

Further comparative analysis of the CAZy database also indicated that the orthologs were significantly separated within groups based on Bray–Curtis non-metric multi-dimensional scaling (NMDS, Figure 5A). After the Dunntest analysis, the TA group showed higher CBM8, GT15, GT74, GH125, GH24, and CBM6 expression levels than those of the TC group, whereas levels of the other 18 enzymes were lower. However, the TQ group showed relatively lower levels of the nine enzymes, including cellulose-binding domain family VIII (CBM8), glycolipid 2-alpha-mannosyltransferase (GT15), and alpha-1,2-L-fucosyltransferase (GT74) than those of the TA group (Figure 5B).

In addition, the relative abundance of the following five enzymes, alpha-1,2-mannosyltransferase (GT87), R. Albus polysaccharide-degrading enzymes (CBM37), rhamnogalacturonan lyase (PL4), 1,2-diacylglycerol 3-beta-galactosyltransferase (GT28), and endo-beta-1,4-galactanase (GH53, Figure 4B) was higher in the TQ group than it was in the TA group. Furthermore, the LDA showed that xylan alpha-1,2-glucuronidase (GH115), exo-alpha-1,6-mannosidase (GH125), and endo-beta-1,4-galactanase (GH53) were dominant enzymes in the TC, TA, and TQ groups, respectively (Figure 5C). Quercetin treatment affected the related enzymes abundance of glycoside hydrolases, glycosyl transferases, polysaccharide lyases, and carbohydrate-binding modules, which facilitated microbial recovery.

Furthermore, the distribution of eggNOG functions was also compared among the three groups, which were clearly separated based on the PCoA (Figure 5D). According to the Dunntest analysis, nine functions (coenzyme transport and metabolism, translation, ribosomal structure and biogenesis, replication, recombination and repair, lipid transport and metabolism, post-translational modification, protein turnover, chaperones, amino acid transport and metabolism, nucleotide transport and metabolism, energy production and conversion, and cell motility) were more disrupted in the TA group than the TC group. However, the TQ group exhibited a higher relative level of these functions, except for cell motility (N), which was lower than that of the other groups (Figure 5E). These results were consistent with those of the KEGG level 2 changes.

**FIGURE 6:**
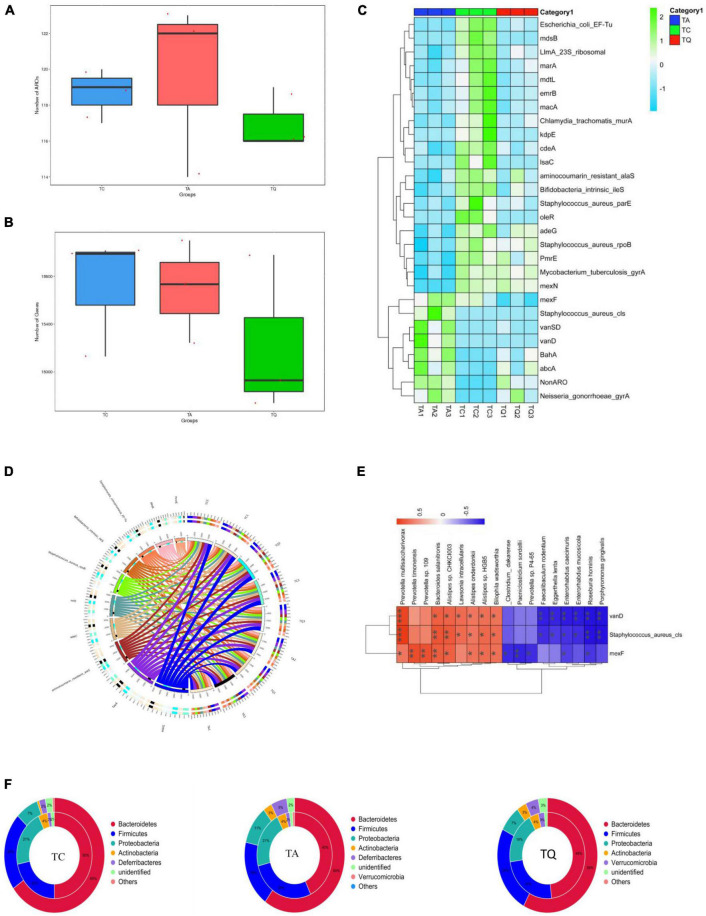
Antibiotic resistance gene annotation on CARD database **(A)**, **(B)** Comparation of gene number and ARO annotation; **(C)** Top 10 AROs detected on samples; **(D)** Significantly different AROs within groups; **(E)** Spearmen correlation analysis of ARO and bacteria species; **(F)** ARO origin analysis at phylum level.

We also analyzed the distribution of antibiotic-resistant genes (ARGs) in the experimental groups. The ARG annotation in the CARD database results showed that 132 antibiotic-resistant organisms (ARO) were annotated, ARO and genes were lowest in the TQ group (Figures 6A,B) and top 10 ARO relative abundance is shown in (Figure 6C). The TA group demonstrated higher levels of *Staphylococcus aureus cls*, *BahA*, *abcA*, *vanSD*, *vanD*, *Neisseria gonorrhoeae gyrA* and *Non-ARO*, compared with TC. However, the TQ group showed significantly lower levels of *S. aureus cls*, *mexF*, and *vanD* than those in the TA group (Figure 6D). The spearmen correlation analysis demonstrated that the above mentioned three genes were negatively related with increased bacteria species in TQ (Figure 6E).

Analysis of the relationship between ARGs, AROs, and species attribution results showed that the proportion of resistance genes from *Proteobacteria* in the TC and TQ groups (7%) was lower than that in the TA group (11%). However, the proportion of resistance genes from *Bacteroidetes* (50% and 48%) was greater than in the TA group (43%), indicating most ARGs in the TQ group found within the *Bacteroidetes* phylum, however, the TA group showed more *Proteobacteria* species (Figure 6F). These results are consistent with the composition of gut microbiota distribution in each group.

Overall, the analysis of the microbiome-associated genes revealed that the TA group displayed a dysfunction in several pathways of the KEGG, CAZy, and eggNOG databases. The metabolism of energy, carbohydrates, lipids, vitamins, glycan, amino acids, and nucleotides was shown to be more disrupted in the TA group than the TC group. However, these metabolic pathways and their related gene expression were regulated in the TQ group, which established a healthy colonic condition.

### Quercetin increased intestinal barrier function

**FIGURE 7:**
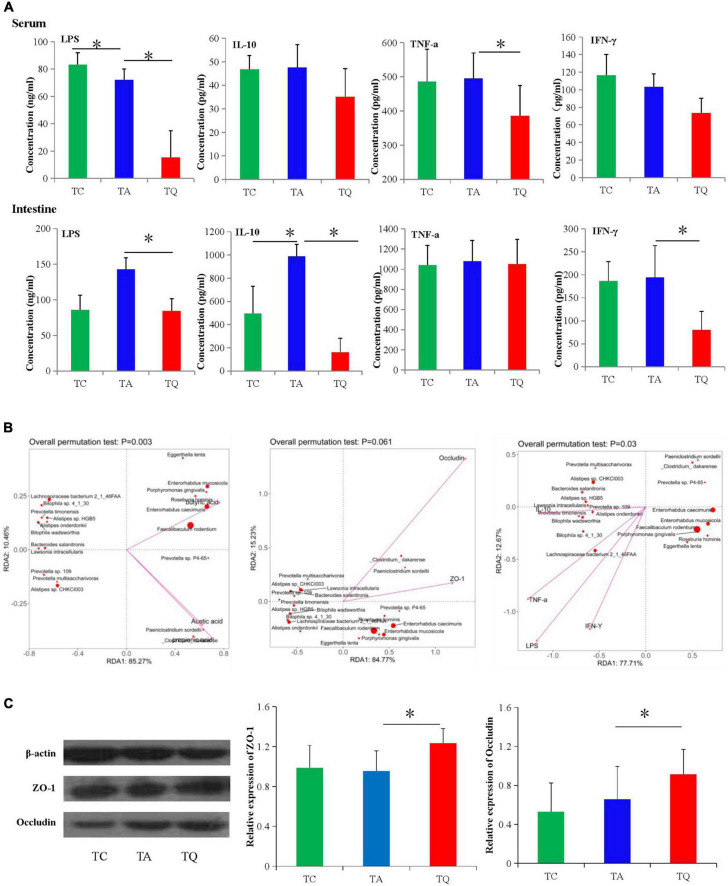
Gut microbial metabolites and gut barrier functional test. **(A)** Serum and intestinal inflammatory index concentration in different groups, *P < 0.05. **(B)** Spearman correlation analysis between significantly different bacteria species and gut barrier indexes. **(C)** Intestinal tight junction proteins (ZO-1 and Occludin) expression test followed by western blot analysis, *P < 0.05.

The intestinal mucosal permeability test in the TQ group showed a significant decrease in serum and large intestinal tissue LPS concentrations (Figure 7A). For the inflammatory indexes, quercetin also significantly decreased the large intestinal IFN-γ, IL-10 and serum TNF-α levels (Figure 7A). Furthermore, these indexes and the microbiota spearman correlation analysis showed that the indexes were positively related to decreased bacteria species in the TQ group, especially *Lachnospiraceae bacterium 2_1_46FAA*, *Alistipes sp. HGB5*, and *Bilophila wadsworthia* (Figure 7B). Moreover, the expression of the intestinal tight junction proteins (ZO-1 and occludin) was significantly higher in the TQ group than the TA group (Figure 7C).

## Discussion

We previously demonstrated that a quercetin-containing diet restored the healthy bacterial composition and structure of antibiotic-treated mice, exhibiting a beneficial prebiotic-like effect, (Shi et al., 2020) and in the present study, our metagenomics analysis showed similar results on the gut microbial structure. In particular, we mainly observed an increase in the relative abundance of butyrate-producing bacterial species including *F. rodentium, E. caecimuris*, *E. mucosicola, A. muciniphila*, and *R. hominis*, which all have a health-promoting effect.

*F. rodentium* is a member of the *Erysipelotrichaceae* family, and it has a high fermentation ability that increases carbohydrate metabolism, produces short-chain fatty acids (SCFA), and exerts anticancer and anti-obesity effects. Consequently, this organism has been hypothesized to mainly replace *Lactobacillu*s and *Bifidobacterium* between the early and late stages of life (Million et al., 2012; Choi et al., 2020). These results showed that quercetin could promote the growth of beneficial bacterial species, while reducing that of pathogenic forms, and thereby reconstruct a healthy gut microbial structure. The increased carbohydrate metabolism, including butanoate metabolism and glycolysis/gluconeogenesis, observed in this study may have been caused by this bacterial species.

*E. caecimuris* has mucosal surfaces colonization and mucin-degrading ability and produces glutamate decarboxylase and arginine dihydrolase (Geurts et al., 2011). *E. caecimuris*, *E. mucosicola*, two bacterial species have high similarity, and we found that they were positively related to butyric acid. Prior research also determined that *E. mucosicola*, has been reported as a bacterial species with 3-ketodeocycholic acid and 12-ketodeocycholic acid deconjugate and dehydrogenate activities (Wegner et al., 2017).

*R. hominis* is a flagellated gut anaerobic bacterium belonging to the *Lachnospiraceae* family in the *Firmicutes* phylum. *R. hominis* has been suggested as a biomarker of health, and was recently shown to exert immunomodulatory properties against gut inflammation by stimulating cytokine gene expression as a toll-like receptor (TLR) agonist (Quan et al., 2018). Patterson et al. determined that *R. hominins* and *Faecalibacterium prausnitzii* were significantly decreased in ulcerative colitis patients, and the presence of *R. hominins* in the gut induced genes involved in promoting the gut barrier function and innate immunity (Machiels et al., 2014; Patterson et al., 2017). Furthermore, this bacteria can penetrate a mucus layer and attach to the surface of the gut epithelial cells of the host, thereby enhancing the probiotic potential (Juge, 2012; Van den Abbeele et al., 2013). In addition, propionate and butyrate, as metabolites of *R. hominins*, promote intestinal melatonin synthesis by promoting 5-hydroxytryptamine (HT) synthesis and activating the phosphorylated-cAMP response element-binding protein (p-CREB)-arylalkylamine *N*-acetyltransferase (AANAT) pathway, thus, offering a potential target for ameliorating intestinal diseases (Song et al., 2021).

*A. muciniphila*, a mucin-degrading bacteria, plays a crucial role in maintaining the mucin layer integrity, thereby reducing translocation of pro-inflammatory LPS and controlling fat storage, adipose tissue metabolism, and glucose homeostasis (Chelakkot et al., 2018). Prior studies have revealed that *A. muciniphila*-derived extracellular vesicles enhanced the expression of occludin and tight junction protein-1, which mediates the negative effects of IFN-γ on glucose tolerance (Greer et al., 2016). In the present study, increased abundance of *A. muciniphila* may reflect a homeostatic response to counteract low-grade inflammation, as evidenced by the decreased serum LPS and IFN-γ in the TQ group.

The normal AIN-93 diet-fed TA group was more highly enriched with *B. wadsworthia*, a saccharolytic intestinal sulfate-reducing bacteria recognized as harmful to colonic epithelial cells (Baron, 1997), than the TQ group was. This bacteria was also shown to be more abundant in carcinoma patients and positively correlated with TNF-α production (Feng et al., 2015). In addition, it promotes Th1-mediated immune response in dietary fat-induced colitis, inhibits butyrate β-oxidation, and degrades butyrate (Muyzer and Stams, 2008). Notably, in this study we observed higher TNF-α levels in the TA group than the TQ group.

Three of the 11 bacterial species with decreased levels in the TQ group belonged to the *Alistipes* genus (*A. onderdonkii*, *Alistipes sp. HGB5*, and *Alistipes sp. CHKCI003*). Species of this genus have been shown to be novel microbial drivers of tumorigenesis in colorectal cancer (Yang and Jobin, 2017) and the ductal microbiome of breast cancer survivors (Chan et al., 2016). In addition, the β-glucuronidase enzymatic activity of these species can lead to carcinogenic toxicity in colorectal cancer (Humblot et al., 2007) and they are associated with diseases of the immune system (Feng et al., 2015; Flemer et al., 2017). These results indicate that the quercetin-supplemented diet facilitated probiotic bacterial growth, which increased gut permeability and decreased the inflammatory index to maintain colonic health. These results also correspond with previous studies showing that prolong treatment with quercetin reduced the levels of the inflammatory markers, IFNγ, TNFα, IL-1, and IL-4 in high-fat diet-induced obese mice (Stewart et al., 2008).

Research on the effects of five other increased bacteria species on the host health are limited. *Prevotella* has been associated with carbohydrate-based diets and degradation of complex polysaccharides, with higher reports among diabetes patients (Mueller et al., 2017). The quercetin treatment increased the prevotella.sp.p4-65stains, but decreased *Prevotella sp. 109*, *Prevotella timonensis*, *Prevotella multisaccharivorax*, and reports related to the characteristic and functions of these remain limited. In addition, a SCFA producing bacteria *Lachnospiraceae bacterium 2_1_46FAA*, which was identified in higher abundance in the healthy subject (Sheng et al., 2021), was decreased, and quercetin selectively increased beneficial bacteria species. *Clostridium darkarense* and *Paeniclostrium*, belonging to *Clostridium spp*., have been reported as strong inducers of colonic T regulatory cell accumulation (Atarashi et al., 2011).

In the TQ group, the dominant bacterial metabolites were SCFA, which can trigger intestinal gluconeogenesis, thereby promoting glucose and energy homeostasis (De Vadder et al., 2014). In particular, butyrate produced by gut bacteria contributes to the gut integrity through the replacement of damaged colon cells and regulation of the expression of tight junction proteins or mucus production (Morrison and Preston, 2016). Increased gut integrity was shown to block LPS leakage into the circulatory system and activation of TLR signaling, which accelerated the release of pro-inflammatory cytokines (Connie et al., 2014). Consistent with this observation, quercetin supplementation decreased serum LPS and increased the expression levels of intestinal tight junction proteins (ZO-1 and occludin).

Previous studies have reported that ZO-1, occludin, and claudin-1 are critical for maintaining the function of the intestinal mucosal mechanical barrier. Our results indicate that during the interaction between quercetin and the gut microbiota, the metabolites play a role as substrates for metabolism or as signaling molecules that could reduce endotoxemia associated with dysbiosis-induced gut barrier damage. These results suggests that quercetin improved microbial metabolic activity and accelerated the metabolism of secondary products, which is consistent with our previous findings of the histological changes of colonic mucosa (Shi et al., 2020). Beyond SCFAs, a number of amino acids, fatty acids, and bile acids are indispensable to the host and they can be provided by bacteria (Metges, 2000). However, further analysis on gut microbial metabolites is required to comprehensively explain the mechanism of quercetin effects on gut permeability.

Although there are some taxonomic variations, the functional capacity of gut microbiota is relatively consistent within the related pathways, gene expression, or co-relation involved in metabolism. A previous study identified the following four butyrate-producing pathways in human gut microbial communities, the acetyl-CoA, glutarate, 4-aminobutyrate, and lysine pathway (Vital et al., 2017). In the present study, the pathway LDA determined lysine biosynthesis to be the predominant pathway in the TQ group, suggesting that quercetin treatment acted through this pathway to increase butyrate production. In addition, the upregulated genes *frdA*, *por*, and *nifJ* could facilitate the TCA cycle (ko00020), pyruvate metabolism (ko00620), and butanoate metabolism (ko00650) through accelerating oxaloacetate to succinate and acetyladenylate to acetate and acetyl-CoA.

Alternatively, these genes could degrade Manolycel CoA, which is related to fatty acid biosynthesis with increased carbohydrate and energy metabolism. Ahn et al. (2008) found that quercetin suppressed lipogenesis by reducing the incorporation rate of fatty acids into adipocyte triacylglycerols in rat fat pads (Motoyashiki et al., 1996) and inhibiting the gene expression levels of fatty acid synthase and the activity of acetyl-CoA carboxylase. In our study, we identified decreased fatty acid biosynthesis in the quercetin treated group, which may be related to increased propionate and decreased acyl carrier protein (K02078) levels. Genes effecting the butyrate production require further validation by RT-PCR.

Tight junctions are formed by a protein complex located at the apical side of epithelial cell membranes. In our study, the quercetin-supplemented diet increased the abundance of tight junction pathway proteins, the KEGG gene *MYH* (k10352), and the expression of the representative tight junction proteins ZO-1 and occludin. This finding indicates that quercetin protected the gut barrier against damage by reducing LPS-induced inflammation. Further, the results demonstrated that quercetin strengthened the intestinal barrier function to prevent the pathogenic bacteria and their metabolites from penetrating the intestinal barrier to affect the health of the host.

The increased cellulose-binding domain family VIII (CBM8), glycolipid 2-alpha-mannosyltransferase (GT15), and alpha-1,2-L-fucosyltransferase (GT74) in TA group reduced by quercetin diets. To obtain more functional insight, a spearman correlation analysis was performed. Results indicated that health beneficial bacteria species were negatively related with CBM8, GT15, GT74 and *Staphylococcus aureus cls*, *mexF*, *vanD*, but positively related to five increased enzymes, alpha-1,2-mannosyltransferase (GT87), R. Albus polysaccharide-degrading enzymes (CBM37), rhamnogalacturonan lyase (PL4), 1,2-diacylglycerol 3-beta-galactosyltransferase (GT28), and endo-beta-1,4-galactanase (GH53) in the TQ group (Supplementary Figure 5 and Figure 6E). The genes and enzyme activity changes were dependent upon bacteria species composition.

In conclusion, the quercetin-supplemented diet accelerated the growth of health beneficial bacteria species (*F. rodentium, E. caecimuris*, *A. muciniphila*, and *R. hominis*), while upregulating genes related to tight junction and energy metabolism and downregulating ARG expression. Butyrate production, which is related to metabolic pathways such as energy metabolism, lysine biosynthesis, and degradation of pathogenic cell motility, and fatty acid biosynthesis, were also positively affected. The KEGG database analysis showed that quercetin exerted significant effects on carbohydrate, energy, and lipid metabolism-related genes and pathways. Finally, our findings might further enhance the understanding of the mechanism of quercetin action and provide a useful foundation for future experimental and clinical studies to work toward its practical application. Further investigation of gut microbial and quercetin metabolites, determination of enzyme activity and gene expression levels are warranted to validate our findings.

## Data availability statement

The data presented in this study are deposited in the NCBI Sequence Read Archive (SRA) under the Bio project number: PRJNA856770, available at: https://www.ncbi.nlm.nih.gov/sra/PRJNA856770.

## Ethics statement

This animal study was reviewed and approved by Institute of Environmental and Operational Medicine.

## Author contributions

TS, CG, and WG conceived, designed, and wrote the manuscript. WM, SZ, LX, and SY executed the laboratory work, and evaluated the data. XB, WL, and ZH pre-processed the sequence data. All authors have contributed significantly to this work and approved the final version of the manuscript.
