# Supplementary material for: Quercetin positively affects gene expression profiles and metabolic pathway of antibiotic-treated mouse gut microbiota
Source: Front Microbiol. 2022 Aug 25;13:983358. doi: 10.3389/fmicb.2022.983358 (PMC9453598; doi:10.3389/fmicb.2022.983358)
Supplement: Supplementary file 1 [file Data_Sheet_1.pdf]

Supplementary figures

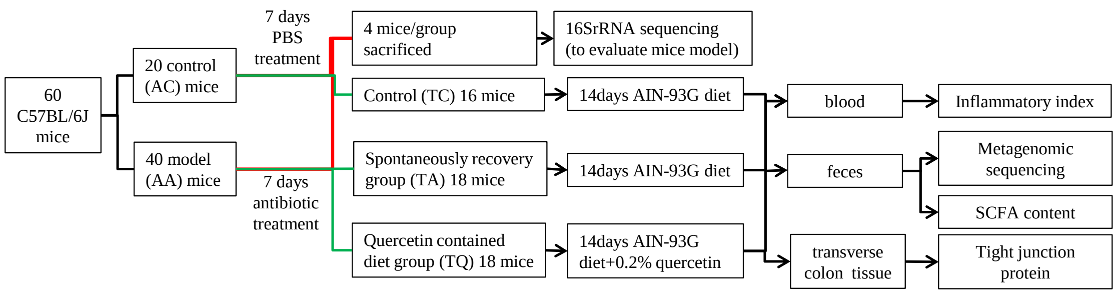

Supplementary figure 1 Chart of experimental protocol

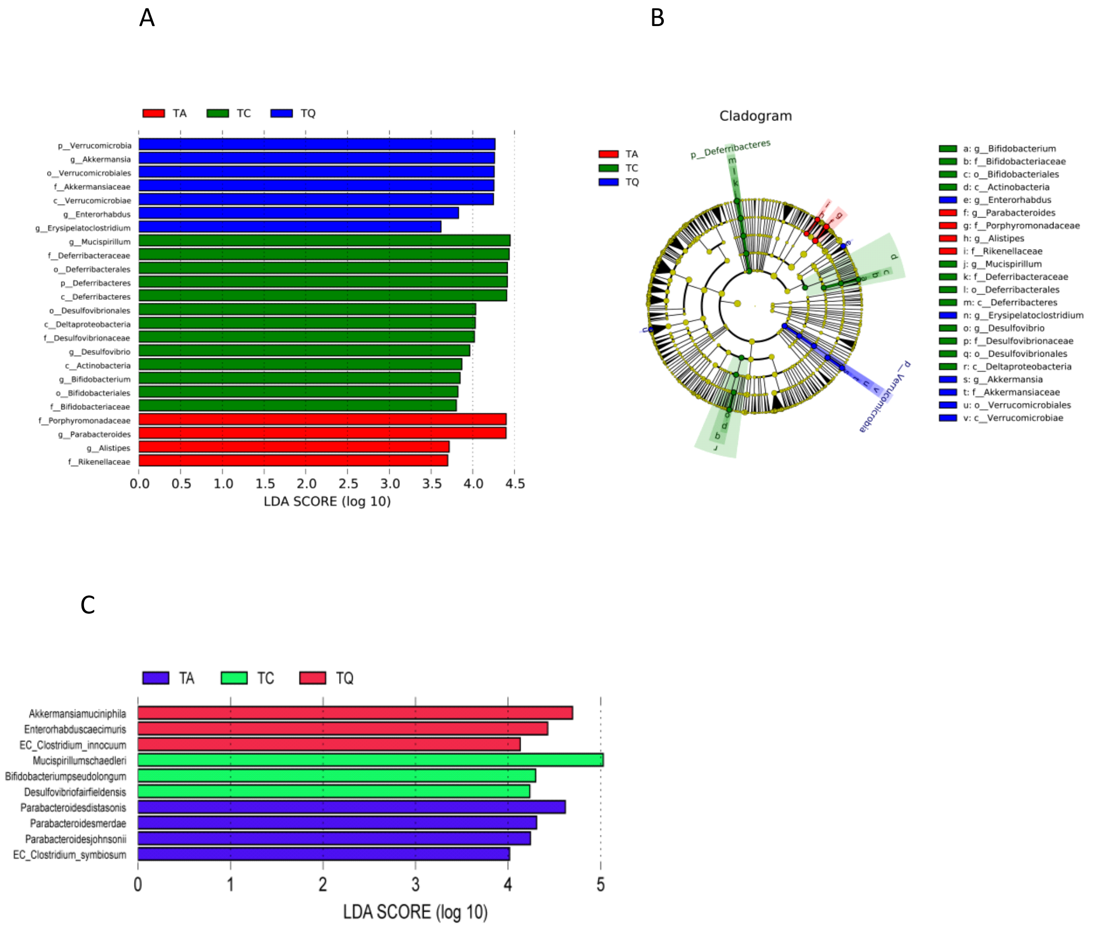

Supplementary figure 2. Gut microbial diversity after quercetin treatment. (A),(B) Bacteria genera Linear Discriminant Analysis (LDA) Effect Size (LEfSe) method using non-parametric factorial Kruskal–Wallis and Wilcoxon rank-sum test. (C), Bacteria species LDA score higher than 4.

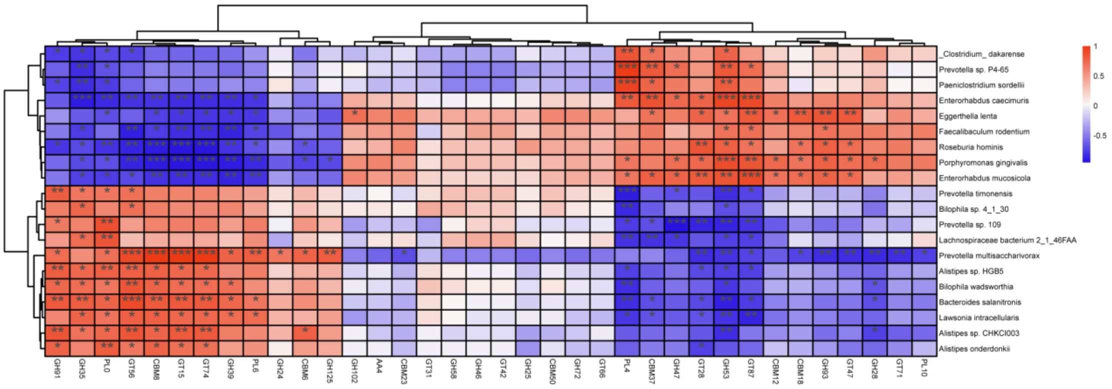

Supplementary figure 3 Significantly different bacteria species and carbohydrate-active enzymes. correlation analysis.

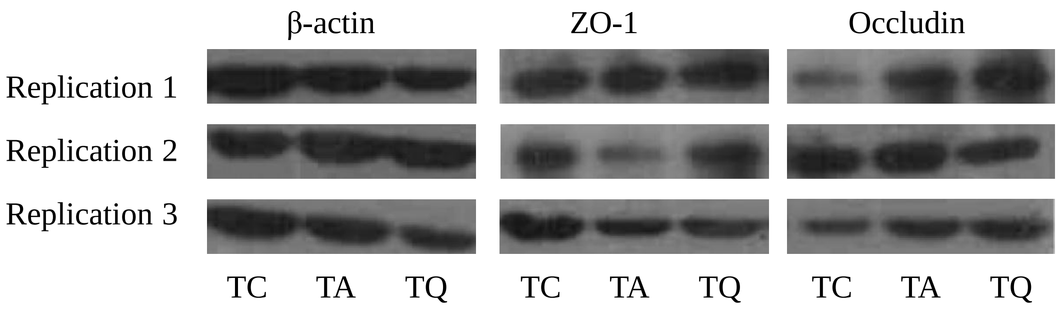

Supplementary figure 4 Gel images of Western blot analysis.
