# Supplementary material for: Quercetin positively affects gene expression profiles and metabolic pathway of antibiotic-treated mouse gut microbiota
Source: Front Microbiol. 2022 Aug 25;13:983358. doi: 10.3389/fmicb.2022.983358 (PMC9453598; doi:10.3389/fmicb.2022.983358)
Supplement: Supplementary file 2 [file Data_Sheet_2.PDF]

Table 1. Significantly different bacteria species between groups.

| Taxonomy (species) | Relative abundance, mean (SD) | | | | P value (Duun test) |
|---|---|---|---|---|---|
| | TA | | TQ | | |
| | Mean | SD | Mean | SD | |
| Faecalibaculum rodentium | 0.027 | 0.017 | 2.777 | 0.742 | 0.038 |
| Enterorhabdus caecimuris | 0.267 | 0.057 | 1.367 | 0.416 | 0.011 |
| Enterorhabdus mucosicola | 0.160 | 0.022 | 0.447 | 0.114 | 0.011 |
| Alistipes sp. CHKCI003 | 0.477 | 0.046 | 0.130 | 0.033 | 0.026 |
| Lachnospiraceae bacterium 2_1_46FAA | 0.267 | 0.021 | 0.110 | 0.028 | 0.038 |
| Bilophila wadsworthia | 0.143 | 0.039 | 0.000 | 0.000 | 0.015 |
| Alistipes sp. HGB5 | 0.063 | 0.012 | 0.033 | 0.005 | 0.017 |
| Bacteroides salanitronis | 0.067 | 0.009 | 0.030 | 0.000 | 0.011 |
| Bilophila sp. 4_1_30 | 0.060 | 0.014 | 0.000 | 0.000 | 0.015 |
| Roseburia hominis | 0.010 | 0.000 | 0.023 | 0.005 | 0.017 |
| Lawsonia intracellularis | 0.027 | 0.005 | 0.007 | 0.005 | 0.026 |
| Prevotella sp. 109 | 0.020 | 0.000 | 0.010 | 0.000 | 0.038 |
| _Clostridium_ dakarense | 0.000 | 0.000 | 0.047 | 0.025 | 0.021 |
| Eggerthella lenta | 0.003 | 0.005 | 0.017 | 0.005 | 0.037 |
| Alistipes onderdonkii | 0.017 | 0.005 | 0.003 | 0.005 | 0.025 |
| Porphyromonas gingivalis | 0.000 | 0.000 | 0.013 | 0.005 | 0.010 |
| Prevotella timonensis | 0.013 | 0.005 | 0.000 | 0.000 | 0.010 |
| Prevotella sp. P4-65 | 0.000 | 0.000 | 0.013 | 0.005 | 0.025 |
| Paeniclostridium sordellii | 0.000 | 0.000 | 0.013 | 0.005 | 0.025 |
| Prevotella multisaccharivorax | 0.013 | 0.005 | 0.000 | 0.000 | 0.025 |

| Taxomony (species) | Relative abundance, mean (SD) | | | | P value (Dunn test) |
|---|---|---|---|---|---|
| | TC | | TA | | |
| | Mean | SD | Mean | SD | |
| Parabacteroides distasonis | 1.707 | 0.097 | 3.237 | 0.175 | 0.017 |
| Parabacteroides merdae | 0.723 | 0.210 | 1.460 | 0.057 | 0.017 |
| Parabacteroides johnsonii | 0.483 | 0.160 | 1.083 | 0.073 | 0.017 |
| Parabacteroides goldsteinii | 0.503 | 0.054 | 0.780 | 0.036 | 0.011 |
| Bifidobacterium pseudolongum | 1.033 | 0.452 | 0.043 | 0.047 | 0.011 |
| Eubacterium plexicaudatum | 0.853 | 0.373 | 0.163 | 0.034 | 0.026 |
| Bacteroides sp. 2_1_33B | 0.277 | 0.009 | 0.527 | 0.038 | 0.017 |
| Bacteroides cellulosilyticus | 0.183 | 0.038 | 0.287 | 0.024 | 0.026 |
| Parabacteroides sp. D26 | 0.147 | 0.005 | 0.307 | 0.025 | 0.011 |
| _Clostridium_ symbiosum | 0.023 | 0.005 | 0.443 | 0.223 | 0.017 |
| Dorea sp. CAG_317 | 0.417 | 0.045 | 0.060 | 0.008 | 0.017 |
| Porphyromonas sp. 31_2 | 0.120 | 0.008 | 0.230 | 0.014 | 0.011 |
| Parabacteroides sp. D13 | 0.123 | 0.012 | 0.223 | 0.012 | 0.017 |
| Bacteroides timonensis | 0.123 | 0.017 | 0.190 | 0.014 | 0.026 |
| Parabacteroides sp. HGS0025 | 0.103 | 0.021 | 0.193 | 0.017 | 0.017 |
| Bacteroides gallinarum | 0.097 | 0.029 | 0.177 | 0.005 | 0.017 |
| Parabacteroides gordonii | 0.087 | 0.017 | 0.180 | 0.014 | 0.011 |
| Parabacteroides merdae CAG_48 | 0.080 | 0.022 | 0.183 | 0.009 | 0.011 |
| Parabacteroides johnsonii CAG_246 | 0.063 | 0.021 | 0.143 | 0.012 | 0.011 |
| Bacteroides sp. 3_1_19 | 0.057 | 0.005 | 0.133 | 0.012 | 0.038 |
| Parabacteroides sp. CAG_2 | 0.063 | 0.005 | 0.133 | 0.012 | 0.026 |
| _Clostridium_ scindens | 0.147 | 0.005 | 0.063 | 0.012 | 0.026 |
| Parabacteroides sp. CAG_409 | 0.057 | 0.005 | 0.110 | 0.008 | 0.011 |
| Gabonibacter massiliensis | 0.050 | 0.008 | 0.083 | 0.005 | 0.017 |

| | | | | | |
|---|---|---|---|---|---|
| Parabacteroides sp. 20_3 | 0.030 | 0.008 | 0.090 | 0.014 | 0.017 |
| Dorea sp. CAG_105 | 0.110 | 0.008 | 0.023 | 0.005 | 0.017 |
| Clostridiales bacterium VE202-16 | 0.017 | 0.005 | 0.100 | 0.043 | 0.017 |
| Shigella flexneri | 0.070 | 0.024 | 0.023 | 0.005 | 0.026 |
| Bifidobacterium sp. AGR2158 | 0.080 | 0.037 | 0.003 | 0.005 | 0.011 |
| Coriobacteriaceae bacterium CHKCI002 | 0.047 | 0.005 | 0.013 | 0.005 | 0.038 |
| Bifidobacterium choerinum | 0.063 | 0.026 | 0.003 | 0.005 | 0.011 |
| Actinobacteria bacterium UC5.1-1B11 | 0.063 | 0.026 | 0.003 | 0.005 | 0.011 |
| Bifidobacterium animalis | 0.057 | 0.025 | 0.003 | 0.005 | 0.011 |
| Lachnospiraceae bacterium 5_1_57FAA | 0.033 | 0.005 | 0.017 | 0.005 | 0.026 |
| Rikenella microfusus | 0.047 | 0.005 | 0.013 | 0.005 | 0.026 |
| Bifidobacterium cuniculi | 0.057 | 0.025 | 0.003 | 0.005 | 0.016 |
| Clostridiales bacterium VE202-26 | 0.033 | 0.005 | 0.013 | 0.005 | 0.017 |
| Parabacteroides sp. ASF519 | 0.013 | 0.005 | 0.030 | 0.000 | 0.017 |
| Paludibacter propionicigenes | 0.030 | 0.000 | 0.013 | 0.005 | 0.017 |
| Parabacteroides sp. D25 | 0.010 | 0.000 | 0.027 | 0.005 | 0.038 |
| Bacteroidales bacterium Barb4 | 0.010 | 0.000 | 0.020 | 0.000 | 0.038 |
| Clostridium bornimense | 0.027 | 0.009 | 0.000 | 0.000 | 0.025 |
| Clostridium sp. CAG_557 | 0.027 | 0.012 | 0.000 | 0.000 | 0.025 |
| Desulfovibrio alaskensis | 0.020 | 0.008 | 0.000 | 0.000 | 0.025 |
| Bifidobacterium gallicum | 0.013 | 0.005 | 0.000 | 0.000 | 0.025 |
| Prevotella multisaccharivorax | 0.000 | 0.000 | 0.013 | 0.005 | 0.025 |
