# Supplementary material for: Quercetin positively affects gene expression profiles and metabolic pathway of antibiotic-treated mouse gut microbiota
Source: Front Microbiol. 2022 Aug 25;13:983358. doi: 10.3389/fmicb.2022.983358 (PMC9453598; doi:10.3389/fmicb.2022.983358)
Supplement: Supplementary file 3 [file Data_Sheet_3.PDF]

Table 2 Metagenomic sequencing data description.

	**TC**	**TA**	**TQ**	**unigene**
PF data	8.58x107±0.51x10^{7}	8.41x107±1.60x10^{7}	8.29x107±1.29x10^{7}	
Clean data	3.22x107±0.05x10^{7}	3.39x107±0.02x10^{7}	3.29x107±0.08x10^{7}	
Gene total sequences	2.03x105±0.47x10^{5}	2.15x105±0.30x10^{5}	2.0x105±0.42x10^{5}	611264
Total bases(bp)	1.04x108±0.58x10^{7}	1.02x108±1.13x10^{7}	0.99x108±0.60x10^{7}	267958446
Average_length(bp)	511.36±10.81	475.65±3.77	485.29±9.47	438.37
N50(bp)	787±37.03	705±3	734±19.97	612
